# Carvedilol dihydrogen phosphate hemihydrate: a powder study

**DOI:** 10.1107/S1600536809029353

**Published:** 2009-07-29

**Authors:** Vladimir V. Chernyshev, Alexandre A. Machula, Sergei Yu. Kukushkin, Yurii A. Velikodny

**Affiliations:** a Department of Chemistry, Moscow State University, 119991 Moscow, Russian Federation; b ‘BION Ltd’, 109 km. Kiev Highway, Obninsk 249032, Kaluga Region, Russian Federation

## Abstract

In the cation of the title compound [systematic name: 3-(9*H*-carbazol-4-yl­oxy)-2-hydr­oxy-*N*-[2-(2-methoxy­phen­oxy)eth­yl]propan-1-aminium dihydrogen phosphate hemihydrate], C_24_H_27_N_2_O_4_^+^·H_2_PO_4_^−^·0.5H_2_O, the mean planes of the tricyclic ring system and the benzene ring form a dihedral angle of 87.2 (2)°. In the crystal structure, the solvent water mol­ecule is situated on a twofold rotation axis linking two cations *via* O—H⋯O and N—H⋯O hydrogen bonds. The anions contribute to the formation O—H⋯O and N—H⋯O hydrogen bonds between the anions and cations, which consolidate the crystal packing.

## Related literature

For the synthesis of the title compound, claimed as Form I, see: Brook *et al.* (2005 ▶). For the crystal structures of two polymorphs of the carvedilol free base, see: Chen *et al.* (1998 ▶); Yathirajan *et al.* (2007 ▶). For details of the indexing algorithm, see: Visser (1969 ▶). The methodology of bond-restrained Rietveld refinement used in this study was the same as described by Chernyshev *et al.* (2003 ▶).
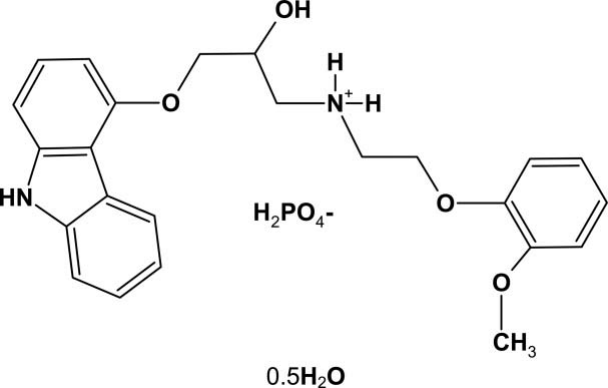


## Experimental

### 

#### Crystal data

C_24_H_27_N_2_O_4_^+^·H_2_PO_4_^−^·0.5H_2_O
*M* *_r_* = 513.47
Monoclinic, 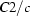

*a* = 26.600 (2) Å
*b* = 12.3767 (12) Å
*c* = 16.5101 (15) Å
β = 106.662 (11)°
*V* = 5207.2 (8) Å^3^
*Z* = 8
Cu *K*α_1_ radiation
μ = 1.38 mm^−1^
*T* = 295 K
Specimen shape: flat sheet
15 × 1 × 1 mm
Specimen prepared at 101 kPa
Specimen prepared at 295 K
Particle morphology: no specific habit, light grey

#### Data collection

Guinier G670 image plate camera
Specimen mounting: thin layer in the specimen holder of the camera
Specimen mounted in transmission mode
Scan method: continuous
2θ_min_ = 5.0, 2θ_max_ = 75.0°
Increment in 2θ = 0.01°

#### Refinement

*R*_p_ = 0.026
*R*_wp_ = 0.035
*R*_exp_ = 0.014
*R*_B_ = 0.064
*S* = 2.43
Wavelength of incident radiation: 1.54059 Å
Excluded region(s): none
Profile function: split-type pseudo-Voigt (Toraya, 1986 ▶)
1346 reflections
157 parameters
125 restraints
H-atom parameters not refined
Preferred orientation correction: March-Dollase (Dollase, 1986 ▶); direction of preferred orientation 100, texture parameter *r* = 1.038 (5)

### 

Data collection: *G670 Imaging Plate Guinier Camera Software* (Huber, 2002 ▶); cell refinement: *MRIA* (Zlokazov & Chernyshev, 1992 ▶); data reduction: *G670 Imaging Plate Guinier Camera Software*; method used to solve structure: simulated annealing (Zhukov *et al.*, 2001 ▶); program(s) used to refine structure: *MRIA*; molecular graphics: *PLATON* (Spek, 2009 ▶); software used to prepare material for publication: *MRIA* and *SHELXL97* (Sheldrick, 2008 ▶).

## Supplementary Material

Crystal structure: contains datablocks I, global. DOI: 10.1107/S1600536809029353/lh2866sup1.cif

Rietveld powder data: contains datablocks I. DOI: 10.1107/S1600536809029353/lh2866Isup2.rtv

Additional supplementary materials:  crystallographic information; 3D view; checkCIF report

## Figures and Tables

**Table 1:** Hydrogen-bond geometry (Å, °)

*D*—H⋯*A*	*D*—H	H⋯*A*	*D*⋯*A*	*D*—H⋯*A*
N19—H19*A*⋯O32	0.90	2.06	2.93 (2)	165
N19—H19*B*⋯O36	0.90	2.18	3.04 (2)	159
N9—H9⋯O35^i^	0.86	1.87	2.72 (3)	168
O18—H18⋯O32^ii^	0.82	2.42	3.15 (2)	148
O18—H18⋯O35^ii^	0.82	2.46	3.02 (2)	126
O33—H33⋯O35^ii^	0.82	1.77	2.53 (2)	153
O34—H34⋯O32^iii^	0.82	1.87	2.58 (2)	144
O36—H36⋯O22	0.85	2.34	2.887 (15)	122
O36—H36⋯O29	0.85	2.00	2.80 (2)	155
C21—H21*B*⋯O34^iii^	0.97	2.24	2.91 (2)	125

Symmetry codes: (i) 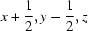; (ii) 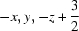; (iii) 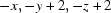.

**Fig. 1.**
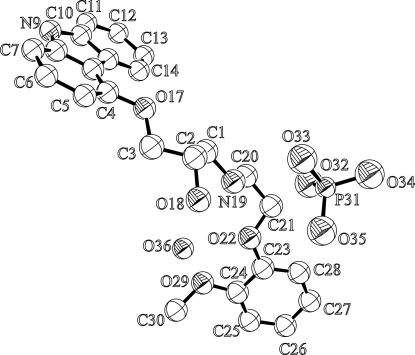
The molecular structure of (I) with the atomic numbering and 40% displacement spheres. H atoms are not shown.

**Fig. 2.**
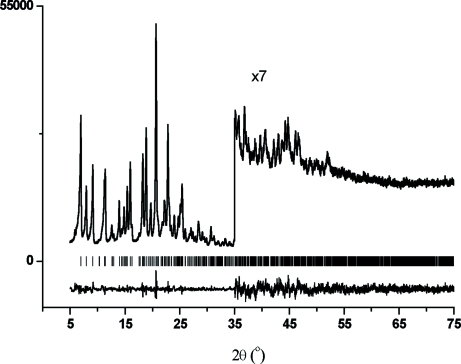
The Rietveld plot, showing the observed and difference profiles for (I). The reflection positions are shown above the difference profile.

## supplementary crystallographic information

### Comment

Earlier, the crystal structures of two polymorphs of carvedilol free base have been reported (Chen *et al.*, 1998; Yathirajan *et al.*, 2007). Herein we report the crystal structure of the title compound (I), also known as carvedilol dihydrogen phosphate hemihydrate, Form I (Brook *et al.*, 2005).

In (I) (Fig. 1), all bond lengths and angles in the cation are comparable with those reported earlier for two monoclinic polymorphs of carvedilol free base (Chen *et al.*, 1998; Yathirajan *et al.*, 2007). The mean planes of tricycle and benzene ring form a dihedral angle of 87.2 (2)°. The crystalline water molecule is situated on a twofold rotational axis linking two cations *via* O—H···O and N—H···O hydrogen bonds (Table 1). The anions contribute to formation O—H···O and N—H···O hydrogen bonds (Table 1) between the anions and cations giving rise to three-dimensional hydrogen-bonding network.

### Experimental

The title compound was synthesized in accordance with the known procedure, invented by Brook *et al.* (2005) for Form I.

### Refinement

During the exposure, the specimen was spun in its plane to improve particle statistics. The monoclinic unit-cell dimensions were determined with the indexing program ITO (Visser, 1969), *M*_20_=35, using the first 30 peak positions. The space group *C*2/*c* was chosen on the basis of systematic extinction rules and confirmed later by the crystal structure solution. The structure of (I) was solved by simulated annealing procedure (Zhukov *et al.*, 2001) and refined following the methodology described in details elsewhere (Chernyshev *et al.*, 2003) by the subsequent bond-restrained Rietveld refinement with the program MRIA (Zlokazov & Chernyshev, 1992). All non-H atoms were refined isotropically: two overall *U*_iso_ parameters were refined for the cation, and two *U*_iso_ parameters were refined for the anion - one for P and one for all O atoms. All H atoms were placed in geometrically calculated positions and not refined. The diffraction profiles and the differences between the measured and calculated profiles are shown in Fig. 2.

### Crystal data

**Table:**

C_24_H_27_N_2_O_4_^+^·H_2_PO_4_^−^·0.5H_2_O	*F*(000) = 2168
*M**_r_* = 513.47	*D*_x_ = 1.310 Mg m^−^^3^
Monoclinic, *C*2/*c*	Cu *K*α_1_ radiation, λ = 1.54059 Å
Hall symbol: -C 2yc	µ = 1.38 mm^−^^1^
*a* = 26.600 (2) Å	*T* = 295 K
*b* = 12.3767 (12) Å	Particle morphology: no specific habit
*c* = 16.5101 (15) Å	light grey
β = 106.662 (11)°	flat_sheet, 15 × 1 mm
*V* = 5207.2 (8) Å^3^	Specimen preparation: Prepared at 295 K and 101 kPa
*Z* = 8	

### Data collection

**Table:**

Guinier G670 diffractometer	Data collection mode: transmission
Radiation source: line-focus sealed tube	Scan method: continuous
Curved Germanium (111)	2θ_min_ = 5.00°, 2θ_max_ = 75.00°, 2θ_step_ = 0.01°
Specimen mounting: thin layer in the specimen holder of the camera	

### Refinement

**Table:**

Refinement on *I*_net_	Profile function: split-type pseudo-Voigt (Toraya, 1986)
Least-squares matrix: full with fixed elements per cycle	157 parameters
*R*_p_ = 0.026	125 restraints
*R*_wp_ = 0.035	27 constraints
*R*_exp_ = 0.014	H-atom parameters not refined
*R*_Bragg_ = 0.064	Weighting scheme based on measured s.u.'s
χ^2^ = 5.928	(Δ/σ)_max_ = 0.004
7001 data points	Background function: Chebyshev polynomial up to the 5th order
Excluded region(s): none	Preferred orientation correction: March-Dollase (Dollase, 1986); direction of preferred orientation 100, texture parameter *r* = 1.038(5)

### Fractional atomic coordinates and isotropic or equivalent isotropic displacement parameters (Å^2^)

**Table:**

	*x*	*y*	*z*	*U*_iso_*/*U*_eq_	
C1	0.1090 (10)	0.7439 (16)	0.8662 (14)	0.096 (9)*	
H1A	0.1218	0.6723	0.8585	0.115*	
H1B	0.1339	0.7771	0.9146	0.115*	
C2	0.1044 (9)	0.8122 (14)	0.7871 (12)	0.096 (9)*	
H2	0.1110	0.8888	0.8012	0.115*	
C3	0.1405 (11)	0.7694 (13)	0.7371 (13)	0.096 (9)*	
H3A	0.1289	0.6980	0.7153	0.115*	
H3B	0.1386	0.8167	0.6895	0.115*	
C4	0.2317 (10)	0.7415 (15)	0.7495 (11)	0.078 (7)*	
C5	0.2335 (8)	0.7825 (16)	0.6713 (14)	0.078 (7)*	
H5	0.2069	0.8273	0.6402	0.094*	
C6	0.2761 (11)	0.7553 (13)	0.6398 (12)	0.078 (7)*	
H6	0.2745	0.7766	0.5851	0.094*	
C7	0.3201 (9)	0.6987 (17)	0.6859 (15)	0.078 (7)*	
H7	0.3502	0.6922	0.6684	0.094*	
C8	0.3156 (9)	0.6522 (16)	0.7609 (13)	0.078 (7)*	
N9	0.3515 (8)	0.5885 (13)	0.8183 (10)	0.078 (7)*	
H9	0.3797	0.5622	0.8107	0.094*	
C10	0.3351 (9)	0.5734 (15)	0.8899 (14)	0.078 (7)*	
C11	0.3572 (11)	0.5123 (16)	0.9629 (15)	0.078 (7)*	
H11	0.3890	0.4766	0.9703	0.094*	
C12	0.3304 (12)	0.5062 (17)	1.0244 (15)	0.078 (7)*	
H12	0.3454	0.4682	1.0741	0.094*	
C13	0.2814 (10)	0.5561 (14)	1.0128 (12)	0.078 (7)*	
H13	0.2646	0.5522	1.0550	0.094*	
C14	0.2580 (9)	0.6116 (13)	0.9379 (14)	0.078 (7)*	
H14	0.2243	0.6393	0.9280	0.094*	
C15	0.2854 (11)	0.6256 (15)	0.8773 (13)	0.078 (7)*	
C16	0.2737 (10)	0.6768 (16)	0.7952 (12)	0.078 (7)*	
O17	0.1930 (7)	0.7639 (11)	0.7893 (9)	0.078 (7)*	
O18	0.0509 (8)	0.7940 (12)	0.7362 (9)	0.096 (9)*	
H18	0.0479	0.8342	0.6957	0.144*	
N19	0.0560 (8)	0.7353 (13)	0.8820 (11)	0.096 (9)*	
H19A	0.0435	0.8022	0.8851	0.115*	
H19B	0.0337	0.7015	0.8379	0.115*	
C20	0.0576 (10)	0.6750 (16)	0.9617 (14)	0.096 (9)*	
H20A	0.0807	0.7124	1.0098	0.115*	
H20B	0.0718	0.6032	0.9592	0.115*	
C21	0.0031 (11)	0.6657 (15)	0.9737 (12)	0.096 (9)*	
H21A	0.0051	0.6343	1.0283	0.115*	
H21B	−0.0133	0.7362	0.9701	0.115*	
O22	−0.0260 (7)	0.5974 (11)	0.9071 (8)	0.096 (9)*	
C23	−0.0747 (10)	0.5587 (16)	0.9079 (13)	0.096 (9)*	
C24	−0.0925 (12)	0.4644 (14)	0.8587 (14)	0.096 (9)*	
C25	−0.1401 (9)	0.4201 (15)	0.8616 (15)	0.096 (9)*	
H25	−0.1524	0.3577	0.8309	0.115*	
C26	−0.1699 (10)	0.4678 (16)	0.9099 (12)	0.096 (9)*	
H26	−0.2032	0.4412	0.9062	0.115*	
C27	−0.1502 (9)	0.5542 (17)	0.9631 (14)	0.096 (9)*	
H27	−0.1677	0.5796	1.0006	0.115*	
C28	−0.1033 (11)	0.6023 (14)	0.9590 (12)	0.096 (9)*	
H28	−0.0911	0.6640	0.9907	0.115*	
O29	−0.0584 (8)	0.4261 (12)	0.8162 (9)	0.096 (9)*	
C30	−0.0784 (11)	0.3728 (16)	0.7357 (14)	0.096 (9)*	
H30A	−0.0497	0.3514	0.7150	0.144*	
H30B	−0.1008	0.4215	0.6961	0.144*	
H30C	−0.0981	0.3101	0.7424	0.144*	
P31	−0.0045 (5)	1.0448 (8)	0.8733 (7)	0.063 (6)*	
O32	0.0094 (8)	0.9380 (12)	0.9182 (10)	0.124 (11)*	
O33	0.0409 (8)	1.0822 (11)	0.8416 (11)	0.124 (11)*	
H33	0.0373	1.0524	0.7960	0.186*	
O34	−0.0127 (8)	1.1325 (13)	0.9331 (10)	0.124 (11)*	
H34	−0.0244	1.1016	0.9678	0.186*	
O35	−0.0536 (9)	1.0330 (11)	0.7999 (9)	0.124 (11)*	
O36	0.0000	0.5716 (11)	0.7500	0.076 (7)*	
H36	−0.0103	0.5310	0.7836	0.114*	

### Geometric parameters (Å, °)

**Table:**

P31—O35	1.51 (2)	C12—C13	1.40 (4)
P31—O34	1.52 (2)	C13—C14	1.40 (3)
P31—O33	1.52 (2)	C14—C15	1.41 (3)
P31—O32	1.509 (18)	C15—C16	1.45 (3)
O17—C4	1.40 (3)	C20—C21	1.52 (4)
O17—C3	1.42 (3)	C23—C28	1.40 (3)
O18—C2	1.45 (3)	C23—C24	1.42 (3)
O22—C23	1.38 (3)	C24—C25	1.39 (4)
O22—C21	1.43 (2)	C25—C26	1.41 (3)
O29—C24	1.38 (3)	C26—C27	1.39 (3)
O29—C30	1.44 (3)	C27—C28	1.40 (4)
O18—H18	0.82	C1—H1A	0.97
O33—H33	0.82	C1—H1B	0.97
O34—H34	0.82	C2—H2	0.98
O36—H36	0.85	C3—H3A	0.97
O36—H36^i^	0.85	C3—H3B	0.97
N9—C10	1.39 (3)	C5—H5	0.93
N9—C8	1.38 (3)	C6—H6	0.93
N19—C1	1.51 (3)	C7—H7	0.93
N19—C20	1.50 (3)	C11—H11	0.93
N9—H9	0.86	C12—H12	0.93
N19—H19A	0.90	C13—H13	0.93
N19—H19B	0.90	C14—H14	0.93
C1—C2	1.53 (3)	C20—H20B	0.97
C2—C3	1.53 (3)	C20—H20A	0.97
C4—C16	1.41 (3)	C21—H21B	0.97
C4—C5	1.40 (3)	C21—H21A	0.97
C5—C6	1.42 (4)	C25—H25	0.93
C6—C7	1.39 (3)	C26—H26	0.93
C7—C8	1.40 (3)	C27—H27	0.93
C8—C16	1.42 (4)	C28—H28	0.93
C10—C15	1.43 (4)	C30—H30A	0.96
C10—C11	1.40 (3)	C30—H30B	0.96
C11—C12	1.40 (4)	C30—H30C	0.96
			
O34—P31—O35	109.7 (13)	O29—C24—C25	128.2 (18)
O32—P31—O35	110.0 (11)	C24—C25—C26	121.3 (19)
O32—P31—O33	109.2 (13)	C25—C26—C27	121 (2)
O32—P31—O34	111.5 (11)	C26—C27—C28	118 (2)
O33—P31—O34	106.4 (12)	C23—C28—C27	120.8 (19)
O33—P31—O35	110.0 (12)	N19—C1—H1B	109.77
C3—O17—C4	117.0 (16)	N19—C1—H1A	109.78
C21—O22—C23	120.0 (18)	H1A—C1—H1B	108.18
C24—O29—C30	120 (2)	C2—C1—H1A	109.73
C2—O18—H18	103.23	C2—C1—H1B	109.71
P31—O33—H33	106.53	C1—C2—H2	111.50
P31—O34—H34	105.88	C3—C2—H2	111.46
H36—O36—H36^i^	107.52	O18—C2—H2	111.57
C8—N9—C10	110 (2)	O17—C3—H3A	109.57
C1—N19—C20	113.1 (18)	C2—C3—H3A	109.48
C10—N9—H9	125.12	C2—C3—H3B	109.48
C8—N9—H9	125.17	O17—C3—H3B	109.59
C1—N19—H19B	108.95	H3A—C3—H3B	108.18
H19A—N19—H19B	107.79	C4—C5—H5	120.24
C20—N19—H19B	108.92	C6—C5—H5	120.11
C1—N19—H19A	108.90	C7—C6—H6	118.16
C20—N19—H19A	109.00	C5—C6—H6	118.07
N19—C1—C2	109.6 (19)	C6—C7—H7	122.62
C1—C2—C3	111.1 (17)	C8—C7—H7	122.59
O18—C2—C1	103.5 (18)	C10—C11—H11	120.69
O18—C2—C3	107.3 (16)	C12—C11—H11	120.78
O17—C3—C2	110.5 (16)	C11—C12—H12	119.21
O17—C4—C16	116.1 (17)	C13—C12—H12	119.26
C5—C4—C16	118 (2)	C12—C13—H13	120.07
O17—C4—C5	125.8 (19)	C14—C13—H13	120.07
C4—C5—C6	120 (2)	C13—C14—H14	120.01
C5—C6—C7	123.8 (19)	C15—C14—H14	119.97
C6—C7—C8	115 (2)	C21—C20—H20A	109.42
N9—C8—C16	108.4 (18)	C21—C20—H20B	109.44
C7—C8—C16	123 (2)	H20A—C20—H20B	107.96
N9—C8—C7	128 (2)	N19—C20—H20A	109.38
N9—C10—C11	131 (2)	N19—C20—H20B	109.33
N9—C10—C15	108.6 (18)	O22—C21—H21A	110.63
C11—C10—C15	121 (2)	C20—C21—H21B	110.63
C10—C11—C12	119 (2)	O22—C21—H21B	110.54
C11—C12—C13	122 (2)	C20—C21—H21A	110.55
C12—C13—C14	120 (2)	H21A—C21—H21B	108.76
C13—C14—C15	120 (2)	C26—C25—H25	119.37
C10—C15—C16	106 (2)	C24—C25—H25	119.29
C14—C15—C16	135 (2)	C25—C26—H26	119.69
C10—C15—C14	119.1 (19)	C27—C26—H26	119.59
C4—C16—C8	120.1 (18)	C26—C27—H27	120.79
C4—C16—C15	133 (2)	C28—C27—H27	120.75
C8—C16—C15	107 (2)	C27—C28—H28	119.57
N19—C20—C21	111.3 (19)	C23—C28—H28	119.65
O22—C21—C20	105.7 (18)	O29—C30—H30B	109.47
O22—C23—C28	123.0 (19)	O29—C30—H30C	109.44
C24—C23—C28	121 (2)	H30A—C30—H30C	109.53
O22—C23—C24	116 (2)	H30B—C30—H30C	109.45
C23—C24—C25	117 (2)	H30A—C30—H30B	109.43
O29—C24—C23	114 (2)	O29—C30—H30A	109.50

Symmetry codes: (i) −*x*, *y*, −*z*+3/2.

### Hydrogen-bond geometry (Å, °)

**Table:**

*D*—H···*A*	*D*—H	H···*A*	*D*···*A*	*D*—H···*A*
N19—H19A···O32	0.90	2.06	2.93 (2)	165
N19—H19B···O36	0.90	2.18	3.04 (2)	159
N9—H9···O35^ii^	0.86	1.87	2.72 (3)	168
O18—H18···O32^i^	0.82	2.42	3.15 (2)	148
O18—H18···O35^i^	0.82	2.46	3.02 (2)	126
O33—H33···O35^i^	0.82	1.77	2.53 (2)	153
O34—H34···O32^iii^	0.82	1.87	2.58 (2)	144
O36—H36···O22	0.85	2.34	2.887 (15)	122
O36—H36···O29	0.85	2.00	2.80 (2)	155
C21—H21B···O34^iii^	0.97	2.24	2.91 (2)	125

Symmetry codes: (ii) *x*+1/2, *y*−1/2, *z*; (i) −*x*, *y*, −*z*+3/2; (iii) −*x*, −*y*+2, −*z*+2.
